# Temple Syndrome: Comprehensive Clinical Study in Genetically Confirmed 60 Japanese Patients

**DOI:** 10.1210/clinem/dgae883

**Published:** 2024-12-18

**Authors:** Tomoe Ogawa, Hiromune Narusawa, Keisuke Nagasaki, Rika Kosaki, Yasuhiro Naiki, Michihiko Aramaki, Keiko Matsubara, Akie Nakamura, Maki Fukami, Tsutomu Ogata, Masayo Kagami

**Affiliations:** Department of Molecular Endocrinology, National Research Institute for Child Health and Development, Tokyo 157-8535, Japan; Department of Advanced Pediatric Medicine, Tohoku University School of Medicine, Tokyo, 157-8535, Japan; Department of Molecular Endocrinology, National Research Institute for Child Health and Development, Tokyo 157-8535, Japan; Department of Pediatrics, Faculty of Medicine, University of Yamanashi, Chuo 409-3898, Japan; Division of Pediatrics, Department of Homeostatic Regulation and Development, Niigata University Graduate School of Medical and Dental Sciences, Niigata 951-8510, Japan; Department of Medical Genetics, National Center for Child Health and Development, Tokyo 157-8535, Japan; Division of Endocrinology and Metabolism, National Center for Child Health and Development, Tokyo 157-8535, Japan; Department of Molecular Endocrinology, National Research Institute for Child Health and Development, Tokyo 157-8535, Japan; Department of Molecular Endocrinology, National Research Institute for Child Health and Development, Tokyo 157-8535, Japan; Division of Diversity Research, National Research Institute for Child Health and Development, Tokyo 157-8535, Japan; Department of Molecular Endocrinology, National Research Institute for Child Health and Development, Tokyo 157-8535, Japan; Department of Molecular Endocrinology, National Research Institute for Child Health and Development, Tokyo 157-8535, Japan; Division of Diversity Research, National Research Institute for Child Health and Development, Tokyo 157-8535, Japan; Department of Molecular Endocrinology, National Research Institute for Child Health and Development, Tokyo 157-8535, Japan; Departments of Pediatrics and Biochemistry, Hamamatsu University School of Medicine, Hamamatsu 431-3192, Japan; Department of Pediatrics, Hamamatsu Medical Center, Hamamatsu 432-8580, Japan; Department of Molecular Endocrinology, National Research Institute for Child Health and Development, Tokyo 157-8535, Japan

**Keywords:** Temple syndrome, clinical findings, diagnostic implication, long-term course, therapeutic implication

## Abstract

**Objective:**

Temple syndrome (TS14) is a rare 14q32.2-related imprinting disorder. Here we report comprehensive clinical findings in TS14.

**Methods:**

We obtained detailed clinical findings in 60 Japanese patients with genetically confirmed TS14, using a questionnaire to attending physicians. The 60 patients consisted of 31 with maternal uniparental disomy 14 [UPD(14)mat], 22 with epimutation, 5 with deletions, and 2 with UPD(14)mat or epimutation.

**Results:**

Small for gestational age, postnatal (∼2 years of age) short stature, and central precocious puberty (CPP) were identified in 88.3%, 87.0%, and 86.0% of patients, respectively. GH therapy was performed in 32 patients, increasing the median height SD score for height from −3.4 to −2.4, and GnRH analog therapy was performed in 32 patients, ameliorating CPP. Furthermore, the survey showed intellectual and developmental disabilities in 21.6% of patients, neurodevelopmental disorders in 21.6% of patients, obesity in 20.0% of patients, hypercholesterolemia in 26.5% of patients aged ≥6 years, diabetes mellitus in 12.8% of patients aged ≥9 years, and Silver-Russell syndrome-like and/or Prader-Will syndrome-like phenotypes in 87.7% of patients in infancy. Notably, 42.9% of patients were enrolled in special classes in childhood, whereas 98.2% of patients attended college or had jobs in adulthood. Hypercholesterolemia and diabetes mellitus were observed before the development of obesity in a substantial fraction of TS14 patients and were controlled by oral medications in most affected patients.

**Conclusion:**

These results clarify the detailed clinical characteristics of TS14. On the basis of these findings, we propose an efficient diagnostic approach and pertinent clinical management for TS14 patients.

Temple syndrome (TS14) (OMIM #616222) is a rare 14q32.2-related imprinting disorder (ID) (1, 2). It is frequently associated with small for gestational age (SGA) and postnatal short stature (SS) plus Silver-Russell syndrome (SRS)-like and/or Prader-Will syndrome (PWS)-like features such as relative macrocephaly, protruding forehead, feeding difficulty, and muscular hypotonia in infancy and central precocious puberty (CPP) in a later age (1-5). TS14 is also often accompanied by placental hypoplasia, psychomotor developmental delay, and metabolic complications such as truncal obesity, hypercholesterolemia, and diabetes mellitus (DM) (1-5).

The diagnosis of TS14 is made when (epi)genetic aberrations are identified in the chromosome 14q32.2 imprinted region [the structure and the character of this imprinted region are shown in Supplementary Fig. S1 (6)]. Indeed, TS14 is caused by maternal uniparental disomy 14 (UPD(14)mat), epimutation (hypomethylation) affecting the normally methylated *MEG3/DLK1*:intergenic-differential methylated region (DMR) and *MEG3*:transcription start site-DMR of paternal origin, and deletions involving the paternally inherited 14q32.2 imprinted region (1, 2). In this regard, it is known that *DLK1* is the causative gene for CPP and is involved in body growth, muscular development, and metabolic homeostasis (7-9) and that *RTL1* is relevant to the body and placental growth and muscular development (10, 11). Thus, while loss of *DLK1* expression would be the major underlying factor for the development of the TS14 phenotype, compromised *RTL1* expression would also be involved in the phenotypic development. Indeed, deletions or sequence variants affecting *DLK1* alone have been classified as the cause for CPP rather than for TS14 (12).

Despite such progress, several matters remain to be clarified with respect to clinical findings in TS14. In particular, long-term growth and maturation patterns including the effects of GH and GnRH analog (GnRHa) therapies and the details of psychomotor developmental delay and metabolic complications remain largely unknown, as well as adulthood phenotype. In addition, *DIO3* involved in the inactivation of thyroid hormones may preferentially be expressed from the paternal allele (13), and *Dio3* knockout mice manifest thyrotoxicosis, color blindness, and hearing loss (14, 15). However, thyroid hormone profile and ophthalmic and auditory functions have not been examined in TS14 patients.

Here we report comprehensive clinical findings in 60 patients with genetically confirmed TS14 and provide useful implications for the diagnosis and management of TS14.

## Patients and Methods

### Ethical Approval

This study was approved by the Institutional Review Board Committee at the National Center for Child Health and Development and was conducted after obtaining written informed consent from the patients and/or their parents.

### TS14 Patients

**Figure 1.**
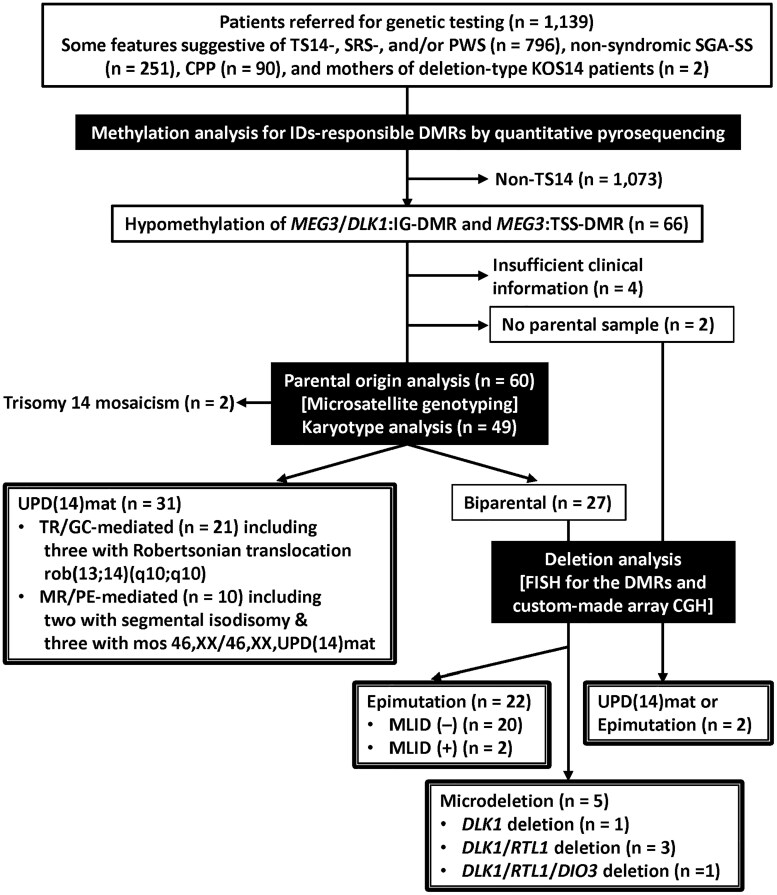
(Epi)genetic diagnostic flow utilized in this study. The following examinations have been performed sequentially, using peripheral blood samples: (1) methylation analysis for multiple ID-related DMRs on various chromosomes by quantitative pyrosequencing analysis, (2) parental origin analysis by microsatellite genotyping for nine loci widely dispersed on chromosome 14 and karyotype analysis, and (3) deletion analysis by fluorescence in situ hybridization for the *MEG3/DLK1*:IG-DMR and *MEG3*:TSS-DMR and custom-made dense array comparative genomic hybridization for the chromosome 14q32.2 imprinted region (Agilent Technologies, Santa Clara, CA). The methods have been reported previously, together with the probe and primer information (2). Abbreviations: DMR, differentially methylated region; ID, imprinting disorder; IG-DMR, intergenic-differential methylated region; KOS14, Kagami-Ogata syndrome; MLID, multilocus imprinting disturbance; MR/PE, monosomy rescue or postfertilization mitotic error; TR/GC, trisomy rescue or gamete complementation; TSS-DMR, transcription start site-differential methylated region; TS14, Temple syndrome; UPD, uniparental disomy.

This study was performed for 60 genetically confirmed Japanese TS14 patients, including the previously reported 32 patients (2), who were identified from a total of 1139 patients referred to us for (epi)genetic diagnosis of IDs [796 with some features suggestive of TS14, SRS, and/or PWS, 251 with nonsyndromic SGA-SS, 90 with CPP, and 2 mothers of deletion-type Kagami-Ogata syndrome (KOS14) patients]. The genetic diagnostic flow employed in this study is shown in Fig. 1. The 60 patients consisted of (1) 31 patients with UPD(14)mat (51.7%) [21 with trisomy rescue or gamete complementation (TR/GC)-mediated UPD(14)mat including 3 patients with Robertsonian translocations rob(13;14)(q10;q10) and 10 with monosomy rescue or postfertilization mitotic error (MR/PE)-mediated UPD(14)mat including 2 patients with segmental isodisomy and 3 patients with 46,XX/46,XX,UPD(14)mat mosaicism]; (2) 22 patients with epimutation (36.7%) [20 without multilocus imprinting disturbance (MLID) for the ID-related DMRs and 2 with MLID including 1 with a homozygous *ZNF445* pathogenic variant (16)]; (3) 5 patients with deletions (8.3%) (1 with a deletion including *DLK1* alone; 3 with deletions encompassing *DLK1* and *RTL1*; and 1 with a deletion spanning *DLK1, RTL1*, and *DIO3*); and (4) 2 patients with UPD(14)mat or epimutation (3.3%) (unclassified due to lack of parental samples).

### Clinical Studies

We obtained comprehensive clinical findings using a questionnaire to attending physicians. Placental weight was assessed using gestational age-matched Japanese references (17). Length/height, weight, occipitofrontal circumference, body mass index (BMI), bone age, pubertal tempo, and endocrine and metabolic data were assessed by the age- and sex-matched Japanese references (18-20). Psychomotor development was evaluated by intellectual quotient/developmental quotient (IQ/DQ) obtained using the *Diagnostic and Statistical Manual of Mental Disorders* (fifth edition) method (21) and by clinical assessment of attending physicians or professional clinicians. BMI values of ≥95 percentile and 85 ∼ <95 percentile were regarded as indications of obesity and overweight, respectively (22). The diagnosis of hypercholesterolemia and DM were based on the Japanese guideline for dyslipidemia (https://www.j-athero.org/jp/wp-content/uploads/publications/pdf/GL2022_s/09.pdf) and that for DM (https://www.jds.or.jp/uploads/files/publications/gl2024/01.pdf). Furthermore, we evaluated the presence or absence of Netchine-Harbison clinical scoring system features for SRS (23) and that of clinical features prompting genetic testing for PWS (24). We also asked the physicians to report any clinical findings not covered by the questionnaire.

### Statistical Analysis

The variable was expressed as the median and range, and the frequency was expressed as the number of patients assessed to be positive for each feature over that of patients examined for the feature. Statistical significance of the median and frequency among 3 groups and between 2 groups was examined by the Kruskal-Wallis test and Fisher's exact test and by Mann-Whitney's *U* test and Fisher's exact test, respectively. These statistical analyses were performed for groups containing ≥5 patients. *P* < .05 was considered significant.

## Results

### Overall Clinical Findings

Clinical findings of all patients are summarized in Table 1, and those of each group are shown in Supplementary Table S1 (6). A comparison of clinical features revealed no significant difference among and between UPD(14)mat, epimutation, and deletion groups and between TR/GC-mediated and MR/PE-mediated UPD(14)mat. Thus, we primarily focused on each feature of total patients.

**Table 1.** Clinical findings in 60 Japanese patients with genetically confirmed Temple syndrome

Patients		Metabolic status	
Sex ratio (male:female)	31:29	BMI at the latest examination (percentile)	65.6 (1.9-99.9), *n* = 60
Age at the genetic diagnosis (years)	4.8 (0.2-33.0), *n* = 60	Obesity	12/60 (20%)
Age at the latest examination (years)	11.0 (1.3-57.0), *n* = 60	Age at diagnosis of obesity (years)	9.2 (1.5-22.3), *n* = 10
*Pregnancy and delivery*		Hypercholesterolemia	13/49 (26.5%) (≥6 years)
Gestational age (weeks)	38 (34-43), *n* = 60	Age at diagnosis of hypercholesterolemia (years)	17.5 (6-47), *n* = 11
Premature delivery (<37 weeks)	9/60 (15.0%)	Diabetes mellitus	5/39 (12.8%) (≥9 years)
Placental weight (SDS)	−1.4 (−2.5 to +1.0), *n* = 19	Age at diagnosis of diabetes mellitus (years)	23 (9-30), *n* = 5
Assisted reproductive technology	5/51 (9.8%)	*Thyroid and ophthalmic and auditory functions*	
Paternal age at childbirth (years)	35 (22-48), *n* = 55	Hyperthyroidism (thyrotoxicosis)	0/60 (0%)
Maternal age at childbirth (years)	32 (19-45), *n* = 56	Hypothyroidism	3/59 (5.1%)
*Growth and maturation*		Thyroid tumor	0/60 (0%)
Prenatal growth		Color blindness	*n* = 1
Small for gestational age^*a*^	53/60 (88.3%)	Hearing loss	*n* = 1
Birth length SDS	−1.9 (−4.9 to +1.2), *n* = 57	*Other features*	
Birth weight SDS	−2.9 (−4.8 to +3.5), *n* = 59	Small hands and feet	39/56 (69.6%)
Birth OFC SDS	−1.2 (−4.2 to +0.7), *n* = 55	Irregular or crowded teeth	28/53 (52.8%)
Postnatal growth		Fifth finger clinodactyly	19/56 (33.9%)
Short stature (≤−2.0 SD) at 24 ± 1 months	40/46 (87.0%)	Male genital abnormalities^*b*^	9/29 (31.0%)
Height SDS at 24 ± 1 months	−3.5 (−4.9 to +0.2), *n* = 46	Recurrent otitis media	14/56 (25.0%)
Weight SDS at 24 ± 1 months	−3.6 (−7.4 to +1.4), *n* = 46	Joint hyperextension	15/54 (27.8%)
Patients treated with GH	*n* = 32	Scoliosis	13/56(23.2%)
Height SDS at 24 ± 1 months in GH-treated patients	−3.6 (−4.9 to −0.9), *n* = 28	Cardiac anomaly	*n* = 1
Height SDS at 24 ± 1 months in GH-untreated patients	−2.8 (−4.6 to +0.2), *n* = 18	*SRS: Netchine–Harbison clinical scoring system features*	
Patients reaching near final height	*n* = 20	Score	3 (1-6), *n* = 53
Near final height SDS	−2.3 (−5.5 to −0.7), *n* = 20	Score ≥4^*c*^	33/57 (57.9%)
Near final height SDS in GH-treated patients	−2.4 (−5.5 to −1.3), *n* = 11	Small for gestational age	53/60 (88.3%)
Near final height SDS in GH-untreated patients	−2.1 (−4.7 to −0.7), *n* = 9	Postnatal growth failure^*d*^	45/57 (78.9%)
Pubertal development		Relative macrocephaly at birth^*e*^	35/55 (63.6%)
Patients entering puberty (male:female)	43/60 (71.7%) (19:24)	Protruding forehead	30/58 (51.7%)
Patients with CPP (male:female)	37 (17:20)	Body asymmetry	12/54 (22.2%)
Frequency of CPP	37/43 (86.0%)	Feeding difficulties and/or low BMI	34/55 (61.8%)
Patients treated with GnRHa (male:female)	*n* = 32 (13:19)	*PWS: salient features prompting genetic testing*	
*Psychomotor development*		Hypotonia with poor suck (infancy)	44/57 (77.2%)
Hypotonia/motor development delay	32/52 (61.5%)	Global developmental delay (≥2 years)	26/55 (47.3%)
Age at head control (months)	6 (3-26), *n* = 52	Excessive eating with central obesity (≥6 years)	4/44 (9.1%)
Age at sitting without support (months)	10 (6-36), *n* = 49	Cognitive impairment (≥13 years)	6/25 (24.0%)
Age at walking without support (months)	19 (12-36), *n* = 45	Hypothalamic hypogonadism (≥13 years)	0/24 (0.0%)
Speech delay	27/46 (58.7%)	Behavior problems (≥13 years)	1/21 (4.8%)
Intellectual and developmental disabilities^*f*^	11/51 (21.6%)	*SRS-like and PWS-like phenotypes*	
Neurodevelopmental disorders	11/51 (21.6%)	SRS-like and PWS-like phenotype	27/57 (47.4%)
Special class until high school age	18/42 (42.9%)	SRS-like phenotype only	6/57 (10.5%)
Job or college after high school age	11/12 (98.2%)	PWS-like phenotype only	17/57 (29.8%)
		SGA-SS phenotype (neither SRS nor PWS)	7/57 (12.3%)

The variable is expressed as the median and range, and the frequency is expressed as the number of patients assessed to be positive for each feature over that of patients examined for the feature.

Abbreviations: BMI, body mass index; CPP, central precocious puberty; GnRHa, GnRH analog; OFC, occipitofrontal circumference; PWS, Prader-Willi syndrome; SDS, SD score; SRS, Silver-Russell syndrome.

^*a*^Birth length and/or birth weight ≤−2 SDS of the gestational age- and sex-matched Japanese reference data (http://jspe.umin.jp/medical/keisan.html).

^*b*^Intelligence/developmental quotient (IQ/DQ) < 70 or clinically unequivocal developmental delay.

^*c*^Micropenis and/or cryptorchidism.

^*d*^Clinical diagnosis of SRS is made when a patient exhibits ≥4 of 6 Netchine-Harbison clinical scoring system features.

^*e*^Height at 24 ± 1 months ≤−2 SDS of the normal children or under midparental target height range.

^*f*^OFC at birth ≥1.5 SDS above birth weight and/or length SDS.

### Patient Profile

The sex ratio was about 1:1. The median age at the genetic diagnosis was 4.8 years and that at the latest examination was 11.0 years.

### Pregnancy and Delivery

The median gestational age was 38 weeks, and premature delivery was reported in 15.0% of patients. Placental weight tended to be low with the median value of −1.4 SD. Five of 51 patients (9.8%) were conceived by assisted reproductive technology (ART) including in vitro fertilization, intracytoplasmic sperm injection, and frozen-thawed embryo transfer but not including ovarian stimulation alone. Although parental age at childbirth was similar among UPD(14)mat, epimutation, and deletion groups, maternal childbearing age was significantly higher in TR/GC-mediated UPD(14)mat (median age 36 years) than in other underlying causes (median age 30 years) (*P* = .0039).

### Growth and Maturation

SGA was observed in 88.3% of patients, with less compromised occipitofrontal circumference (OFC), and short stature at ∼2 years of age was found in 87.0% of patients. GH therapy was performed in 32 patients, with a dosage for SGA-SS in 22 patients (0.23-0.47 mg/kg/week) and with that for GH deficiency (GHD) in 10 patients (0.175 mg/kg/week) [Supplementary Table S2 (6)] (for the criteria of GH treatment; the diagnosis of GHD was made after multiple provocation tests in most patients). At the start of GH therapy, the median age was 4.2 years, and the median height was −3.4 SD. Serum IGF-I values were obtained in 31 of the 32 GH-treated patients, and they were below −2.0 SD in 5 of the 21 SGA-SS patients and within the low-normal range in all 10 GHD patients.

Forty-three patients had entered puberty at the time of this survey, and 37 patients (86.0%) showed CPP [for the criteria of CPP diagnosis, see the footnote of Supplementary Table S3 (6)]. At the diagnosis of CPP, the median age was 7.3 years in girls and 8.8 years in boys, and the median height was −1.6 SD in girls and −1.0 SD in boys. Bone ages were advanced by 1.9 years in girls and 1.1 years in boys, and endocrine data were consistent with pubertal entry. GnRHa therapy (30-120 μg/kg/dose, every 4 weeks) was performed in 32 patients (19 girls and 13 boys) shortly after the diagnosis but not performed in the remaining 5 patients (1 girl and 4 boys).

**Figure 2.**
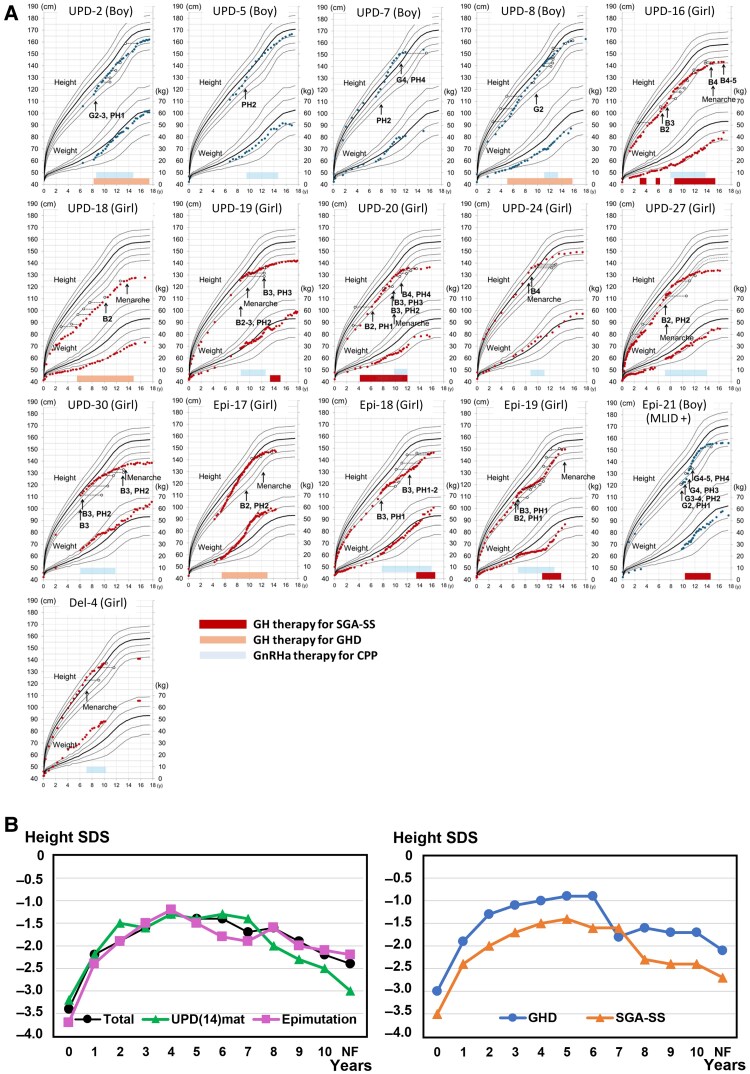
Long-term growth and maturation data. (A) Growth charts in 16 patients with Temple syndrome reaching near final heights. The data are plotted on the growth curves of normal Japanese children (height: +2 SD, +1 SD, the mean, −1 SD, −2 SD, −3 SD; weight: +2 SD, +1 SD, the mean, −1 SD, and −2 SD). (B) Annual change of the median height SDS after GH treatment. Abbreviations: B, breast; CPP, central precocious puberty; G, genitalia; GH, growth hormone; GHD, growth hormone deficiency; GnRHa, gonadotropin-releasing hormone analog; NF, nearly final height age; PH, pubic hair; SDS, standard deviation score; SGA-SS, small for gestational age and short stature; UPD, uniparental disomy.

We collected long-term growth and maturation data, including near final heights (growth rate < 1.0 cm/year) in 20 patients. Longitudinal growth and maturation data in 16 of the 20 patients are shown in Fig. 2A. Growth and maturation patterns were complicated by multiple factors including the degree of SS and/or CPP, the presence or absence of therapeutic intervention, the age at the start of therapeutic intervention (eg, the start of GH therapy before or after pubertal entry and that of GnRHa therapy before or after menarche), and the variable dosages and durations of GH and GnRHa therapy. Despite such complexity, the integrated annual growth data of GH-treated patients revealed that the median height SD score (SDS) gradually increased during the first 4 to 6 years after GH treatment, probably due to the beneficial effects of GH and the influence of CPP, and gradually decreased afterward, probably due to early growth cessation, with a similar pattern between UPD(14)mat and epimutation (Fig. 2B) [Supplementary Table S2 (6)]. Consequently, the median height increased from −3.4 SD to −2.4 SD with GH treatment. Notably, while height SDS at ∼2 years of age was significantly lower in GH-treated patients than in GH-untreated patients (*P* = .012), near final height SDS was similar between the 2 groups. In addition, the median height SDS remained higher in patients treated with GH dosage for GHD than in those treated with GH dosage for SGA-SS from the start of GH therapy [Fig. 2B, Supplementary Table S2 (6)]. Pubertal development progressed rapidly. For example, in 5 girls treated after menarche, the median age at thelarche was 7.5 years (reference age in Japanese girls, 9.6 ∼ 10.0 years) (18) and that at menarche was 8.5 years [reference age in Japanese girls, 12.3 ± 1.25 years (18); thus, the median duration from thelarche to menarche was only 1.0 year] [Supplementary Table S3 (6)]. By contrast, menarche was well controlled with a median age of 13.4 years in 5 girls treated before menarche. Similar findings were also observed in boys, although precise assessment was difficult in boys.

### Psychomotor Development

Hypotonia was observed in 61.5% of patients; speech delay in 58.7% of patients; intellectual and developmental disabilities (IDDs) in 21.6% of patients; and neurodevelopmental disorders (NDDs) such as autism spectrum disorder (ASD), attention deficit hyperactivity disorder (ADHD), and pervasive developmental disorders in 21.6% of patients. In addition, 42.9% of patients were enrolled in special classes until their high school ages, whereas 98.2% of patients attended college or had regular or simple jobs (66.7% of patients attended college or had regular jobs) after their high school ages.

Clinical findings of 21 patients with IDDs and/or NDDs are shown in Supplementary Table S4 (6). Of 18 patients examined for both IDDs and NDDs, 1 patient (5.6%) showed both IDDs and NDDs, 10 patients (55.6%) exhibited IDDs alone, and 7 patients (38.9%) manifested NDDs alone. Most patients attended special classes until their high school ages, whereas 3 of 4 patients had simple jobs and the remaining 1 patient attended college after their high school ages. Some of them showed social withdrawal and/or school refusal.

### Metabolic Status

**Figure 3.**
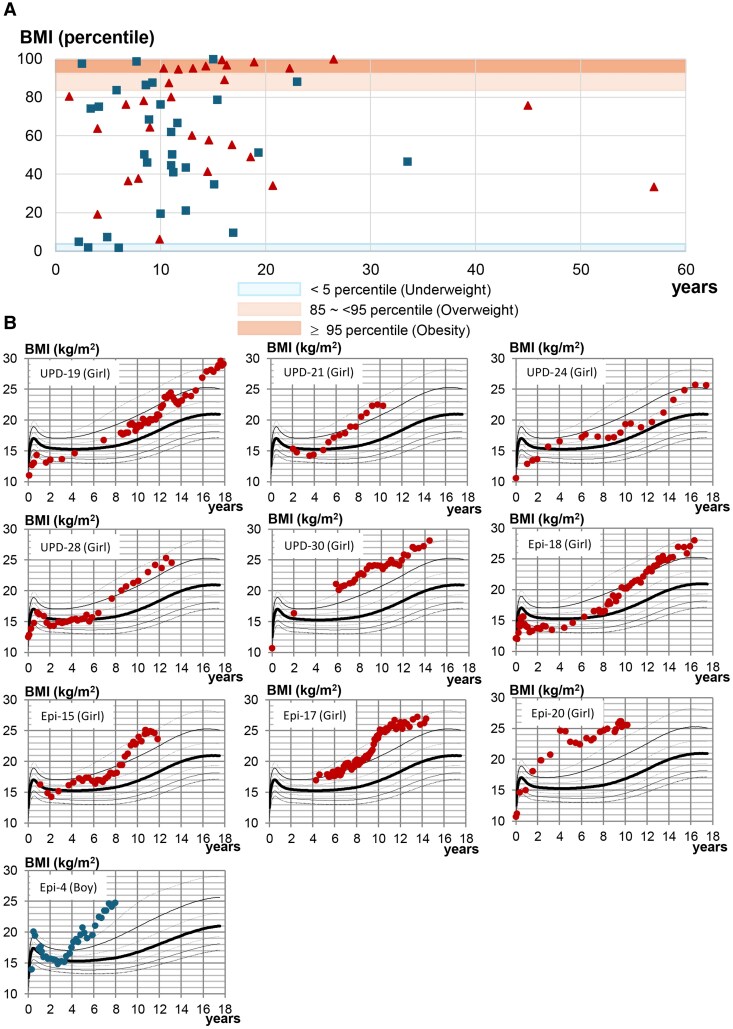
BMI findings (male and female data are shown in square and triangle, respectively). (A) BMI percentiles at the latest examination in 60 patients with Temple syndrome. (B) Longitudinal BMI charts in patients with obesity. The data are plotted on the BMI curves of normal Japanese children (97th, 90th, 75th, 50th, 25th, 10th, and 3rd percentiles). Abbreviations: BMI, body mass index; Epi, epimutation; UPD, uniparental disomy.

BMIs tended to be high with the median values of 65.6% at the latest examination in the 60 patients; 12 patients (20.0%) were classified as obese and 6 patients (10.0%) as overweight (Fig. 3A). The median age at the diagnosis of obesity was 9.2 years. The long-term BMI data in 10 of the 12 patients with obesity is shown in Fig. 3B. Although they were rather thin in infancy, they exhibited truncal obesity from childhood and remained obese afterward, except for a single patient (Epi-20) who became obese from infancy. Hyperphagia was reported in 4 patients from childhood.

Hypercholesterolemia was found in 13 of 49 patients aged ≥ 6 years (26.5%) with the median age at diagnosis of 17.5 years. Of the 13 patients, BMI was obtained in 10 patients, and just 2 of the 10 patients were obese at the time of diagnosis [Supplementary Table S5 (6)]. Four of 8 patients with low-density lipoprotein-hypercholesterolemia received oral medication. Hydroxymethylglutaryl-coenzyme A reductase inhibitors utilized in 2 patients successfully reduced serum low-density lipoprotein cholesterol, although other drugs were apparently ineffective. The remaining 4 patients were under nutritional and exercise management.

DM was reported in 5 of 39 patients aged ≥ 9 years (12.8%), with the median age at diagnosis of 23 years. Three patients were obese at the time of diagnosis [Supplementary Table S5 (6)]. Two patients received insulin therapy, whereas another 2 patients were fairly well treated with oral antidiabetic drugs such as sodium-glucose cotransporter 2 inhibitors (SGLT2) and glucagon-like peptide 1 agonists (1 of the patients required insulin during pregnancy). The remaining 1 patient was under nutritional management.

### Thyroid & Ophthalmic and Auditory Functions

We investigated these features observed in *Dio3* knockout mice [Supplementary Table S6 (6)] (14, 15). Hyperthyroidism (thyrotoxicosis) was absent, whereas mild hypothyroidism, which required short- or long-term levothyroxine treatment, was detected at the neonatal screening in 1 patient and during the follow-up of GH therapy in 2 patients. One male patient had red-green color blindness, and 1 patient had congenital deafness, which required hearing aids. One patient with a deletion including *DIO3* had no such features.

### Adulthood Phenotypes

The data of 12 patients aged ≥18 years (post-high school age) revealed the median height of −2.1 SD, the history of GH treatment in 54.5% of patients, IDDs in 16.7% of patients, NDDs in 25.0% of patients, a median BMI of 22.8, hypercholesterolemia in 63.6% of patients, and overt DM in 41.7% of patients [Supplementary Table S7 (6)]. Most patients attended college or had some regular job.

### Other Features

Small hands and feet and abnormal teeth were observed in >50% of patients, and several minor features were identified in >20% of patients. In addition, ventral septal defect was identified in a single patient.

### SRS-like and PWS-like Features

Thirty-three of 57 patients satisfied the Netchine-Harbison clinical diagnostic criteria for SRS (score ≥ 4/6), with pre-and postnatal growth failure being most frequent, although they tended to show truncal obesity from childhood. Body asymmetry (hemihypoplasia) was often identified in UPD(14)mat and epimutation [Supplementary Table S1 (6)]. Forty-four of 57 patients manifested PWS-like hypotonia with poor suck in infancy, although characteristic PWS features after infancy were infrequent in TS14 patients. Overall, 47.4% of patients showed both SRS-like and PWS-like phenotypes, 10.5% of patients manifested SRS-like phenotype alone, 29.8% of patients exhibited PWS-like phenotype alone, and 12.3% of patients had SGA-SS phenotype (neither SRS nor PWS) in infancy.

## Discussion

We performed a comprehensive clinical study in 60 patients with genetically confirmed TS14. The relative frequency of underlying (epi)genetic causes was grossly comparable to that reported previously (12) and MLID was infrequent as reported previously (2, 25). This would argue for the accuracy of the genetic data in TS14. Furthermore, clinical features were comparable among and between UPD(14)mat, epimutation, and deletion. This would be consistent with loss of *DLK1* and/or *RTL1* expression being the major underlying factor for the development of the TS14 phenotype (7-11).

Pregnancy and delivery were characterized by relative placental hypoplasia. This reflects the critical role of imprinting genes in placental development (26). Notably, 9.8% of patients were conceived by ART. Considering that the median age at the latest examination was 11.0 years in the 60 patients and that the frequency of ART-conceived subjects is ∼4% in the Japanese general population of 11 years ago (https://www.jsog.or.jp/activity/art/2021_JSOG-ART.pdf), this would argue that ART can be a risk factor for the development of TS14 as has been reported in several IDs (27, 28). Furthermore, advanced maternal childbearing age in TR/GC-mediated UPD(14)mat is consistent with the production of disomic oocytes being a maternal age-dependent phenomenon (27).

Growth and maturation studies revealed several critical findings. First, TS14 was frequently associated with pre- and postnatal growth failure. This is compatible with loss of paternally expressed *DLK1* and/or *RTL1* with a growth-promoting effect (26). Second, overall, the height was increased by ∼1.0 SD during GH therapy. This argues for the beneficial effects of long-term GH therapy on statural growth in TS14, as well as short-term GH therapy (29). Third, the median height SDS at ∼2 years of age was lower in GH-treated patients than in GH-untreated patients. This indicates that GH therapy was preferentially performed in patients with severe SS. Fourth, the median height SDS was constantly lower in patients treated for SGA-SS than in those treated for GHD from the start of GH therapy, despite the GH dosage being larger in SGA-SS patients than in GHD patients. This implies that SGA-SS patients have more severely compromised growth potential than GHD patients. Fifth, GHD was diagnosed in 10 patients. It is unlikely, however, that TS14 patients have true GHD, because the diagnosis was made after repeatedly performed GH provocation tests with low accuracy (30), and serum insulin-like growth factor-I (IGF-I) values remained low but within the normal range in GHD patients. Sixth, serum IGF-I was < −2.0 SD in a certain fraction of SGA-SS patients. This would be due to severe undernutrition in such patients (31). Lastly, pubertal development progressed rapidly in patients untreated before menarche in girls, while it was well controlled by GnRHa therapy. This suggests that GnRHa therapy should be started shortly after the pubertal onset in TS14 patients. In addition, GnRHa therapy would also serve to ameliorate SS in patients who have entered puberty from early childhood (eg, before 6 years old in girls) (32). Collectively, although the data were complicated, it appears that the therapeutic interventions served to ameliorate statural growth and pubertal development.

Psychomotor development studies disclosed not only hypotonia and speech delay in ∼60% of patients but also mild IDDs and NDDs in ∼20% of patients. In this regard, previous studies have reported that hypotonia is frequent in TS14 and could underlie the speech delay via impaired ability to move oral structures (33). However, the data on IDDs and NDDs remain small in TS14, although Gillessen et al found mild IDDs in 5 of 8 patients (4) and Juriaans et al identified NDDs in 2 of 15 patients (5). This study indicates that IDDs and/or NDDs represent important features in TS14, although the coexistence of IDDs and NDDs was rare. Indeed, while IDDs and NDDs remain rather infrequent in TS14 patients, their prevalences are higher than that in the general population (∼1% for IDDs and ASD and ∼5% for ADHD) (34, 35).

Metabolic abnormalities were also revealed in TS14. TS14 patients, though thin in infancy, often showed truncal obesity from childhood and remained obese afterward. Furthermore, they frequently developed hypercholesterolemia and/or DM from the late teens to early 20s. Such metabolic abnormalities are reminiscent of those of PWS (24). However, hyperphagia was rare in TS14 patients, and hypercholesterolemia and DM were observed before the development of obesity in a substantial fraction of TS14 patients. Since TS14 is associated with absent expression of *DLK1*, which functions as a negative regulator for adipogenesis and plays an essential role in metabolic homeostasis (7), this may underlie the development of hypercholesterolemia and/or DM in the absence of obesity and hyperphagia in TS14.

Hyperthyroidism was absent, and color blindness was reported only in a single patient as was hearing loss. In addition, 1 patient with a deletion involving *DIO3* had no *DIO3*-related findings. Furthermore, it has been reported that (1) the prevalence of red-green color blindness is 4.7% to 8.0% in Japanese males (36) and that of congenital deafness is ∼0.16% in Japanese subjects (37); (2) KOS14 with 2 copies of *DIO3* of paternal origin have no clinically discernible hypothyroidism (38); and (3) *DIO3* is not imprinted in the placenta (39), although it is somewhat preferentially expressed from the paternal allele in the somatic tissues (13). These findings imply that *DIO3* is not imprinted or just partially imprinted in most tissues and that a certain amount of *DIO3* expression dosage can protect from the development of an abnormal phenotype.

In adulthood, TS14 patients showed grossly sum of the aforementioned phenotype. TS14 is characterized by a tendency of short and obese habitus, IDDs and NDDs, and metabolic complications in adulthood. This implies the importance of long-term periodical health checks for psychomotor and metabolic complications. Notably, most patients went to college or had jobs. This suggests that most adult patients can get on with their social life.

Most patients exhibited SRS- and/or PWS-like phenotypes in infancy, as reported previously (1-5). Thus, SRS- and/or PWS-like phenotype is a key feature in the identification of TS14. Compared to our previous study (2), the relative frequency of the SRS-like phenotype was decreased and that of the PWS-like phenotype was increased. In this regard, the costs for genetic diagnosis and GH therapy for PWS, but not for SRS or TS14, are covered by the national health insurance in Japan. Thus, it is likely that genetic diagnosis is performed for many patients with PWS-like features and that genetically undiagnosed patients have preferentially been referred to our research center. Notably, hemihypoplasia was not as specific to epimutation in contrast to the high prevalence of epimutation (hypomethylation) of the *H19/IGF2*: intergenic-DMR in SRS patients with body asymmetry (23), although the reason remains unknown. Furthermore, most of the minor anomalies identified in >20% of patients are frequently reported in SRS and/or PWS. For example, small hands/feet and scoliosis are frequently found in PWS (24), whereas irregular or crowded teeth, fifth finger clinodactyly, and male genital abnormality are often identified in SRS (23). This would also imply the phenotypic overlap between TS14 and SRS/PWS.

**Figure 4.**
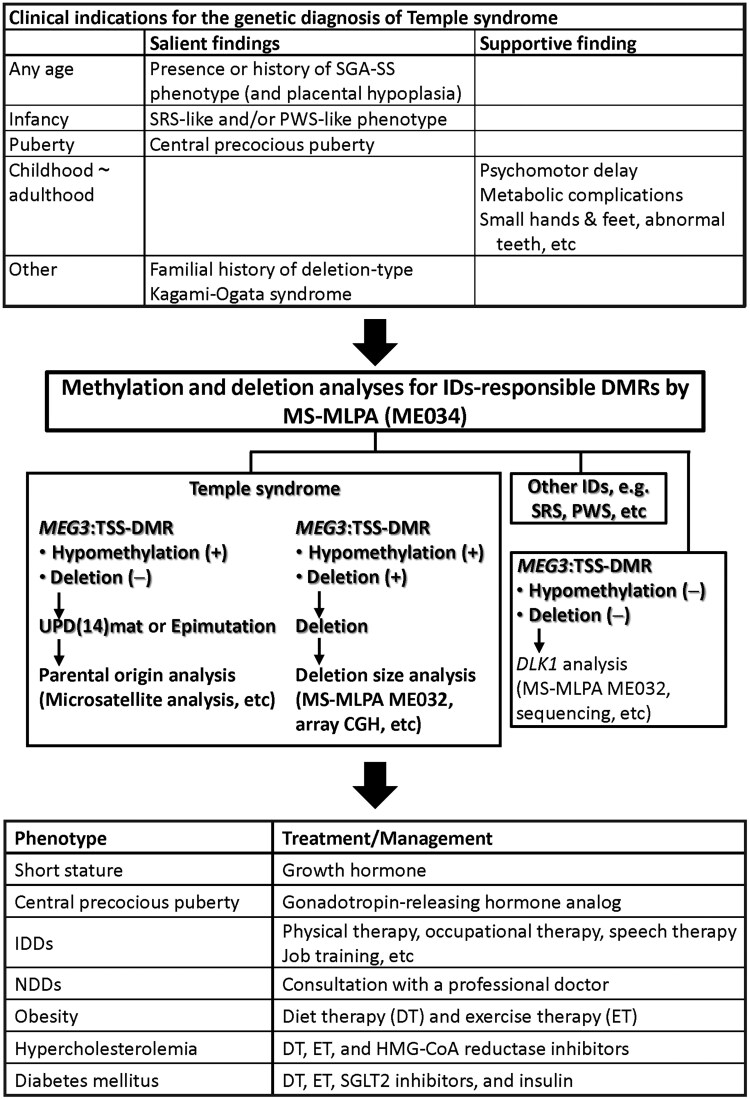
Summary of diagnosis and management of Temple syndrome. MS-MLPA ME034 contains the probe for *MEG3*:TSS-DMR but not that for *MEG3/DLK1*:IG-DMR, whereas MS-MLPA ME032 contains the probes for both *MEG3*:TSS-DMR and *MEG3/DLK1*:IG-DMR. The *MEG3*:TSS-DMR probe can examine both methylation and deletion patterns, whereas the *MEG3/DLK1*:IG-DMR probe can examine deletion pattern alone. Abbreviations: HMG-CoA, 3-hydroxy-3-methylglutaryl-coenzyme A; ID, imprinting disorder; IDD, intellectual and developmental disability; IG-DMR, intergenic-differential methylated region; MS-MLPA, methylation-specific multiplex ligation dependent probe amplification; NDD, neurodevelopmental disorder; PWS, Prader-Willi syndrome; SGA-SS, small for gestational age and short stature; SGLT2, sodium-glucose cotransporter 2 inhibitors; SRS, Silver-Russell syndrome; TSS-DMR, transcription start site-differential methylated region.

This study provides useful implications for the diagnosis of TS14 (Fig. 4). Considering that TS14 is a genetically rather than clinically diagnosed ID (1, 2), we have previously proposed that genetic studies for TS14 should be performed in patients with salient TS14 features, ie, SGA-SS phenotype (and placental hypoplasia) plus SRS-like and/or PWS-like phenotype in infancy and CPP in puberty, as well as a familial history of deletion-type KOS14 (2). This study provides strong support for the appropriateness of this proposal. Furthermore, this study indicates that (1) TS14 patients often show discernible IDDs and/or NDDs and become obese from childhood; (2) hypercholesterolemia and DM frequently develop from the late teens to early 20s; (3) small hands and feet, as well as abnormal teeth, are prevalent from childhood; and [4] TS14 patients usually remain short and obese and often show psychomotor problems and metabolic problems in adulthood. Thus, when these findings are observed in patients with the aforementioned salient features, this would provide further support for the clinical diagnosis of TS14.

For genetic diagnosis, we recommend methylation-specific multiplex ligation dependent probe amplification (MS-MLPA; ME034 Probemix) (MRC-Holland, Amsterdam) as the first-tier testing (Fig. 4). ME034 is widely available and can reveal aberrant methylation patterns and deletions affecting TS14-, SRS-, and PWS-related DMRs at once. Thus, MS-MLPA can not only permit the genetic diagnosis of TS14 and SRS/PWS but also identify deletions that may be present in relatives and may cause TS14 or KOS14 in the offspring depending on the sex of the patients (2, 38). In this regard, we performed quantitative pyrosequencing for IDs-responsible DMRs as the screening for TS14. Indeed, we have performed quantitative pyrosequencing as a routine method for the detection of IDs, because it can quantify the degree of DNA methylation (40). To perform the quantitative pyrosequencing, however, each research laboratory must prepare specialized costly equipment and reagents, and, therefore, there would be some variation among laboratories. Furthermore, quantitative pyrosequencing cannot distinguish deletions from UPD(14)mat and epimutation. Actually, we have reproduced the genetic findings in nine of the 60 patients [3 with UPD(14)mat, 3 with epimutation, and 3 with deletion] using ME034 Probemix for multiple IDs-responsible DMRs on various chromosomes and ME032 Probemix for multiple loci at the imprinted regions on chromosomes 7 and 14. In addition, when no genetic abnormality has been detected, it would be worth examining deletions involving *DLK1* but sparing the DMRs by MS-MLPA (ME032 Probemix) and pathogenic *DLK1* variants by sequencing, because *DLK1* abnormalities often cause TS14-like phenotypes (41).

This study also provides valuable implications for the management of TS14 (Fig. 4). First, GH therapy is suggested to promote statural growth. Second, GnRHa therapy is recommended for CPP. Third, appropriate physical therapy, occupational therapy, speech-language-hearing therapy, and job training should be performed for patients with IDDs to maximize their social abilities, and pertinent consultation with developmental pediatricians and psychiatrists is suggested for patients with NDDs to ease social difficulty. Fourth, diet therapy and exercise therapy are recommended when BMI begins to increase, and medications for hypercholesterolemia such as hydroxymethylglutaryl-coenzyme A reductase inhibitors and those for DM such as SGLT2 and insulin should be utilized for metabolic abnormalities. Importantly, such management should be carried out by a multidisciplinary team, to cover every important issue.

In conclusion, we performed a comprehensive clinical study in 60 genetically diagnosed TS14 patients. The results not only clarify detailed clinical findings such as long-term growth and maturation patterns including the effects of GH and GnRHa therapies and the details of psychomotor developmental delay and metabolic complications as well as adulthood phenotype but also provide useful implications for the diagnosis and management of TS14 patients.

## Data Availability

The data sets generated and analyzed during the present study are not publicly available but are available from the corresponding author on reasonable request.

## Abbreviations

ADHD: attention deficit hyperactivity disorder
ART: assisted reproductive technology
ASD: autism spectrum disorder
BMI: body mass index
CPP: central precocious puberty
DM: diabetes mellitus
DMR: differentially methylated region
GH: growth hormone
GHD: GH deficiency
GnRHa: GnRH analog
HMG: hydroxymethyl glutaryl
ID: imprinting disorder
IDD: intellectual and developmental disability
IGF-I: serum insulin-like growth factor-I
IQ/DQ: intellectual quotient/developmental quotient
KOS14: Kagami-Ogata syndrome
LDL: low density lipoprotein
MLID: multilocus imprinting disturbance
MR/PE: monosomy rescue or postfertilization mitotic error
MS-MLPA: methylation-specific multiplex ligation dependent probe amplification
NDD: neurodevelopmental disorder
OFC: occipitofrontal circumference
PWS: Prader-Will syndrome
SDS: SD score
SGA: small for gestational age
SGLT2: sodium-glucose cotransporter 2 inhibitors
SRS: Silver-Russell syndrome
SS: short stature
TR/GC: trisomy rescue or gamete complementation
TS14: Temple syndrome
UPD(14)mat: maternal uniparental disomy 14
