# Correction to: “Temple Syndrome: Comprehensive Clinical Study in Genetically Confirmed 60 Japanese Patients”

**DOI:** 10.1210/clinem/dgaf398

**Published:** 2025-07-15

In the above-named article by Ogawa T, Narusawa H, Nagasaki K, Kosaki R, Naiki Y, Aramaki M, Matsubara K, Nakamura A, Fukami M, Ogata T, and Kagami M (*J Clin Endo Metab*. 2024; doi: 10.1210/clinem/dgae883), there was an error in the affiliations section.

In the originally published article, in the affiliations section, author Tomoe Ogawa’s affiliations were “^1^Department of Molecular Endocrinology, National Research Institute for Child Health and Development, Tokyo 157-8535, Japan; and ^2^Department of Pediatrics, Tohoku University School of Medicine, Sendai 980-8574, Japan.” In the corrected article, the affiliations have been updated to read, “^1^Department of Molecular Endocrinology, National Research Institute for Child Health and Development, Tokyo, 157-8535, Japan; and ^2^Department of Advanced Pediatric Medicine, Tohoku University School of Medicine, Tokyo, 157-8535, Japan.”

The article has been corrected online.

doi: 10.1210/clinem/dgae883

